# Unveiling green corrosion inhibitor of *Aloe vera* extracts for API 5L steel in seawater environment

**DOI:** 10.1038/s41598-024-64715-z

**Published:** 2024-06-18

**Authors:** Ahmad Royani, Muhammad Hanafi, Nabisab Mujawar Mubarak, Gadang Priyotomo, Victor Sunday Aigbodion, Siti Musabikha, Azwar Manaf

**Affiliations:** 1 Research Center for Metallurgy, National Research and Innovation Agency–BRIN, Kawasan KST B. J. Habibie, Serpong, Tangerang Selatan, Banten 15314 Indonesia; 2 Research Center for Pharmaceutical Ingredients and Traditional Medicine, National Research and Innovation Agency–BRIN, Kawasan KST B. J. Habibie, Serpong, Tangerang Selatan, Banten 15314 Indonesia; 3 Petroleum and Chemical Engineering, Faculty of Engineering, Universiti Teknologi Brunei, Bandar Seri Begawan, BE1410 Brunei Darussalam; 4 Department of Chemistry, School of Chemical Engineering and Physical Sciences, Lovely Professional University, Jalandhar, Punjab India; 5 Department of Metallurgy and Materials Engineering, University of Nigeria, Nsukka, 410001 Nigeria; 6 Faculty of Engineering and the Built Environment, University of Johannesburg, Auckland Park, P. O. Box 534, Johannesburg, South Africa; 7 Postgraduate Program of Materials Science Study, Department of Physics, Faculty of Mathematics and Natural Sciences, Universitas Indonesia, Depok, 16424 Indonesia

**Keywords:** *Aloe vera* extract, API 5L steel, Characterization, Environment media, Green inhibitor, Biochemistry, Biotechnology, Plant sciences, Environmental sciences

## Abstract

This study evaluated *Aloe vera* extract as a green inhibitor to prevent corrosion in seawater environments. *A. vera* extract was produced by maceration with methanol﻿–water at room temperature. Electrochemical techniques were used to evaluate the corrosion inhibitor effectiveness of the *A. vera* extract. The morphology of the corrosion products was analyzed by FE-SEM equipped with EDS and AFM. FT-IR and LCMS characterized the functional and structural groups in this extract. The electrochemical measurements show that *A. vera* extract could effectively reduce the corrosion of API 5L steel in seawater environments. Inhibition efficiency (IE) increases with increasing concentration. Optimal corrosion inhibition efficiency of around 83.75% (PDP) and 88.60% (EIS) was obtained by adding 300 mg L^−1^ of extract at 310 K. Furthermore, the higher the concentration of *A. vera* extract, the greater the activation energy (E_a_), with the highest activation energy being 48.24 kJ mol^−1^ for the concentration of 300 mg L^−1^. Conversely, increasing the temperature and exposure duration reduces the corrosion inhibition efficiency (IE) values; the best exposure period was 30 min with 88.34% IE by a concentration of 300 mg L^−1^ at 300 K. This corrosion inhibition is achieved by the adsorption process of *A. vera* bioactive on metal surfaces with a mixed inhibitor through a physisorption-chemisorption mechanism. This finding was confirmed by the smoother surface morphology of the steel treated with *A. vera* extract than without. This unveiling investigation found that *A. vera* extract has the potential to be an environmentally friendly corrosion inhibitor in the seawater environment.

## Introduction

The corrosion of metallic materials and their alloys in seawater environments is a significant challenge for companies that rely on seawater flows. Integrating offshore infrastructure and its adjacent regions is inherently intertwined with using construction metals, notably iron and steel^1^. Metals such as iron and steel are widely utilized in infrastructure due to their malleability and ease of manufacturing^2^. Iron metal is susceptible to corrosion, particularly in harsh conditions with high sulfide or chloride ion concentrations^3^ and flow rates^4^. Chloride ions can induce localized corrosion, known as pitting corrosion, on the steel surface, leading to material failure and potential accidents^5,6^. Metals and alloys can be safeguarded from corrosion attacks by implementing environmental alteration procedures involving inhibitors. Considering the growing global consciousness surrounding environmental sustainability, there has been a notable surge in scholarly attention towards plant-derived corrosion inhibitors with green properties^7,8^. The exploration of green corrosion inhibitors is now ongoing. It remains a prominent subject in safeguarding various metals and alloys in controlling corrosion in aggressive environments, both acid and alkaline^9–13^.

Environmental corrosion inhibitors derived from plant extracts can be generated from various plant parts, including bark, leaves, roots, and fruit^14–16^. The rise in research on green corrosion inhibitors is closely linked to their properties, which involve active compounds with double bonds and heteroatoms like N, S, O, and OH, which play a crucial role in preventing corrosion^17–20^. In addition, green inhibitors are often regarded as environmentally benign, non-toxic, readily available, straightforward, and cost-effective^21^. Hence, this ecologically sustainable botanical-derived inhibitor exhibits promise as a viable alternative to traditional synthetic corrosion inhibitors, which possess hazardous and toxic properties, are highly costly, non-biodegradable, and hence lack environmental friendliness^21,22^. Several investigations have shown evidence of the utilization of green inhibitors in various media^10,23^ and in different metal applications^9^. Studies conducted on the tomato pomace have demonstrated its capacity to significantly inhibit the corrosion of mild steel in a NaCl environment, with an efficiency level of up to 98%^24^. The investigations regarding the inhibitory effects of zinc cation (Zn^2+^) and *Nepeta pogonosperma* leaf extract at mild steel were also conducted in a 3.5% NaCl medium. The most favorable outcomes were observed using a combination of 300 ppm extract and 700 ppm Zn solution^25^. In addition, it has been shown that watermelon-zinc extract can reduce corrosion in a low-carbon environment using a sodium chloride medium (NaCl 3.5%)^26^. The application of using agricultural food waste as a cost-effective and environmental corrosion inhibitor has also been evaluated for mild steel in a sodium chloride solution^27^. The main components of the extract consist of phenolic and flavonoid compounds, which are strongly suspected to play an essential role in inhibiting the corrosion rate on carbon steel surfaces by atomic interactions and adsorption on metal surfaces^15^.

This work focused on evaluating the environmental corrosion inhibitors from *Aloe vera (A. vera)* on API 5L steel in seawater environments. In this corrosion study, seawater media was selected because two-thirds of the earth's surface area is ocean. Apart from that, seawater is also an aggressive solution because it contains chloride ions, microorganisms, and other corrosive mediums^28–30^. *A. vera* was chosen in this study because apart from having many benefits in various health areas, it is also rich in antioxidants^31–34^. This antioxidant property is critical in preventing corrosion because it forms complex compounds and protects metal surfaces^35^. Several studies of *A. vera* extract reported that the active compounds contained in *A. vera* have the potential to act as corrosion inhibitors in acids^36–41^. However, studies and evaluations of the active substances from *A. vera* extract as inhibitors in the seawater environment are still relatively rare; thus, they should be investigated further.

Therefore, this investigation aimed to analyze *A. vera* extract's performance in developing eco-friendly corrosion inhibitors that are environmentally and easy to implement in seawater environments. Most green inhibitor studies are conducted in acidic or basic conditions; hence, seawater is a novelty in this study. This evaluation ensures that *A. vera*'s potential active compounds are an alternative source to substitute conventional harmful inhibitors. *A. vera* was extracted using a new maceration technique with a reactor in a methanol–water solution. Evaluation of extract performance as inhibitor based on extract concentration, media temperature, and immersion time using electrochemical techniques (open circuit potential, potentiodynamic polarization, and electrochemical impedance spectroscopy) in seawater environment simulated with synthetic seawater media. In addition to efficiency values, the protection effect and surface morphology were also analyzed by FESEM–EDS and AFM. An in-depth analysis of inhibiting mechanism, adsorption, thermodynamics, and kinetics was also described.

## Materials and experimental

### Material and electrolite

API 5L steel from geothermal industrial pipes was used as the working electrode with the main content of nominal chemical composition (wt.%): 96.17% Fe, 0.113% C, 0.288% Si, 1.147% Mn, 0.190% P, 0.063% S, 0.17% Cr, 0.0045% Mo, 0.0025% Ni, 0.0059% Cu, 0.047% Al, 0.025% Pb, 0.016% Ti, and 0.0012% Zn.

The electrolyte solution in this work is a synthetic seawater solution made from a mixture of salt powder (Marine Art SF-1, Japan) and distilled water. The solution is made by dissolving 38-g salt powder for every 1000 mL. The main composition of salt powder consists of 22.1 g NaCl, 9.9 g MgCl⋅6H_2_O, 1.5 g CaCl_2_⋅2H_2_O, 3.9 g Na_2_SO_4_, 0.61 g KCl, and 0.19 g NaHCO_3_.

### Environmentally corrosion inhibitor

The green inhibitor used for API 5L steel corrosion is sourced from *A. vera* extract. The *Aloe vera *(L.)* Burm. f. (Asphodelaceae)* was acquired from the Indonesian Medical and Aromatic Crops Research Institute (BALITTRO), Bogor, West Java, and identified in the Botany Laboratory (Herbarium Bogoriense), Directorate of Scientific Collection Management, National Research and Innovation Agency (BRIN) (Letter ID: B-3369/II.6.2/DI.05.07/9/2022). The extraction procedure was carried out following previous work^42^. Briefly, 25 g of dried *A. vera* was extracted with 200 mL methanol–water (1:1 v/v) for 3 × 24 h at room temperature with a change of new solution every day. Next, it was filtered and evaporated at a temperature of 323 K with a speed of 70 rpm using a vacuum evaporator. The bioactive structure of *A. vera* extract was characterized using liquid chromatography-mass spectrometry-mass spectrometry (LCMS-MS) and Fourier transform infrared spectrometry (FT-IR, Bruker-Tension).

### Electrochemical study

Electrochemical studies were performed with a corrosion measurement system (Gamry, PCI4G750-50090). Before electrochemical measurements, the workpiece specimens were sanded with sandpaper to a size of 1200 mesh and then rinsed with running water and acetone. In this study, measurements were carried out with a conventional three-electrode cell assembly (API 5L steel as the working electrode, graphite as the auxiliary electrode, and calomel as the reference electrode). Before potentiodynamic polarization (PDP) and impedance spectroscopy (EIS) measurements, open circuit potential (OCP) measurements were carried out for 10 min to obtain a stable potential. The potentiodynamic polarization was measured with a scanning rate of 1 mV/s at ± 250 mV versus OCP, and electrochemical parameters (i_corr_, E_corr_, corrosion rate) were determined from the polarization curves using Echem-Analyst software. The inhibition efficiency value (IE, %) was determined using Eq. (1)^43^:

1 $$IE (\%)=\frac{{\left({i}_{corr}\right)}_{o}-{\left({i}_{corr}\right)}_{1}}{{\left({i}_{corr}\right)}_{o}}\times 100$$

where (i_corr_)_1_ represents the corrosion currents with *A. vera* extract, while (i_corr_)_0_ denotes the corrosion currents without extract (mA/cm^2^). Meanwhile, electrochemical impedance spectroscopy (EIS) analysis was utilized at the frequency level of 0.1 Hz to 0.5 MHz using AC with 10 points/decade of resolution. The inhibitor efficiency value (IE, %) with EIS is determined based on Eq. (2) below^44^

2 $$IE\left({\%}\right)=\frac{{R}_{ct 1}-{R}_{ct 0}}{{R}_{ct 1}}\times 100$$

R_ct1_ and R_ct0_ represent polarization resistance with inhibitor and without the addition of inhibitors (blank), respectively. All measurements and evaluations (OCP, PDP, and EIS) were done at least three times to ensure reproducibility and stability of measurements.

### Surface analysis

The steel surface morphology appeared using Field Emission Scanning Electron Microscopy (FE-SEM, Jeol Multibeam JIB-4610F) and energy dispersion spectroscopy (EDS). The surface roughness and morphology of the steel were analyzed using Atomic Force Microscopy (AFM, Park NX10).

## Results and discussion

### Characteristic of *A. vera* extracts

**Figure 1:**
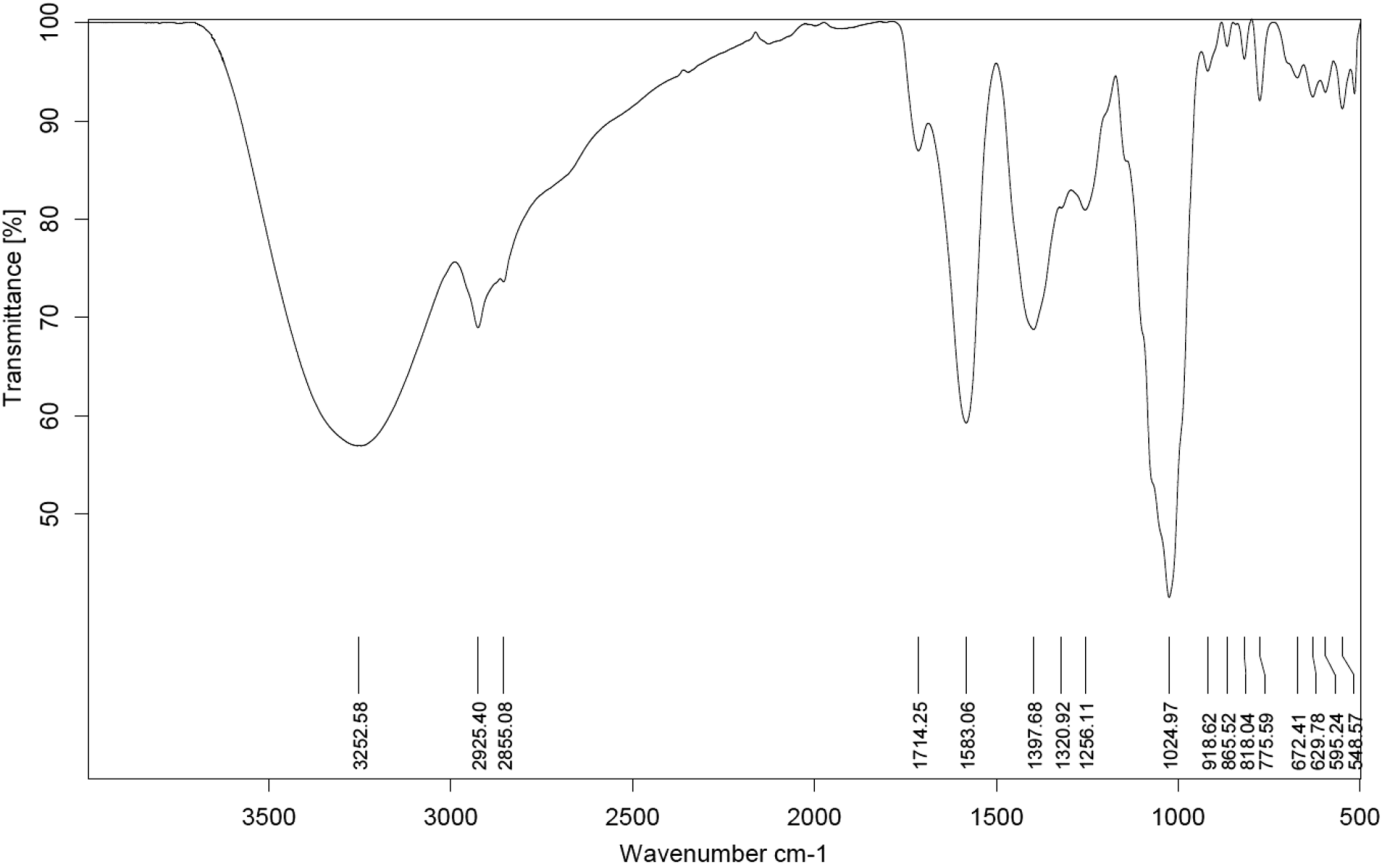
FT-IR results of *A. vera* extracts.

Figure 1 depicts the results of the FT-IR spectrum study conducted on the *A. vera* extract. The infrared spectrum reveals that *A. vera* extract possesses many functional groups, enhancing active chemicals' ability to associate with metal surfaces by adsorption. The main absorption peak at 3252.58 cm^−1^ in *A. vera* extract suggests the existence of OH or NH bonds, indicating the existence of alcohol compounds or amide group chemicals. At a wavelength of 2925.40 cm^−1^, another peak indicates the stretching of C-H bonds, suggesting the presence of hydrocarbon group molecules. Furthermore, the signal peak observed at 1024.97 cm^−1^ indicates the existence of amine chemicals. The signal observed at 1583.06 cm^−1^ predicts the presence of the aromatic compound, namely the stretching of the C=O bond. Furthermore, several peaks at 1397.68 cm^−1^, 1320.92 cm^−1^, and 1256.11 cm^−1^ suggest the potential existence of C–N and C–O functional groups.

**Figure 2:**
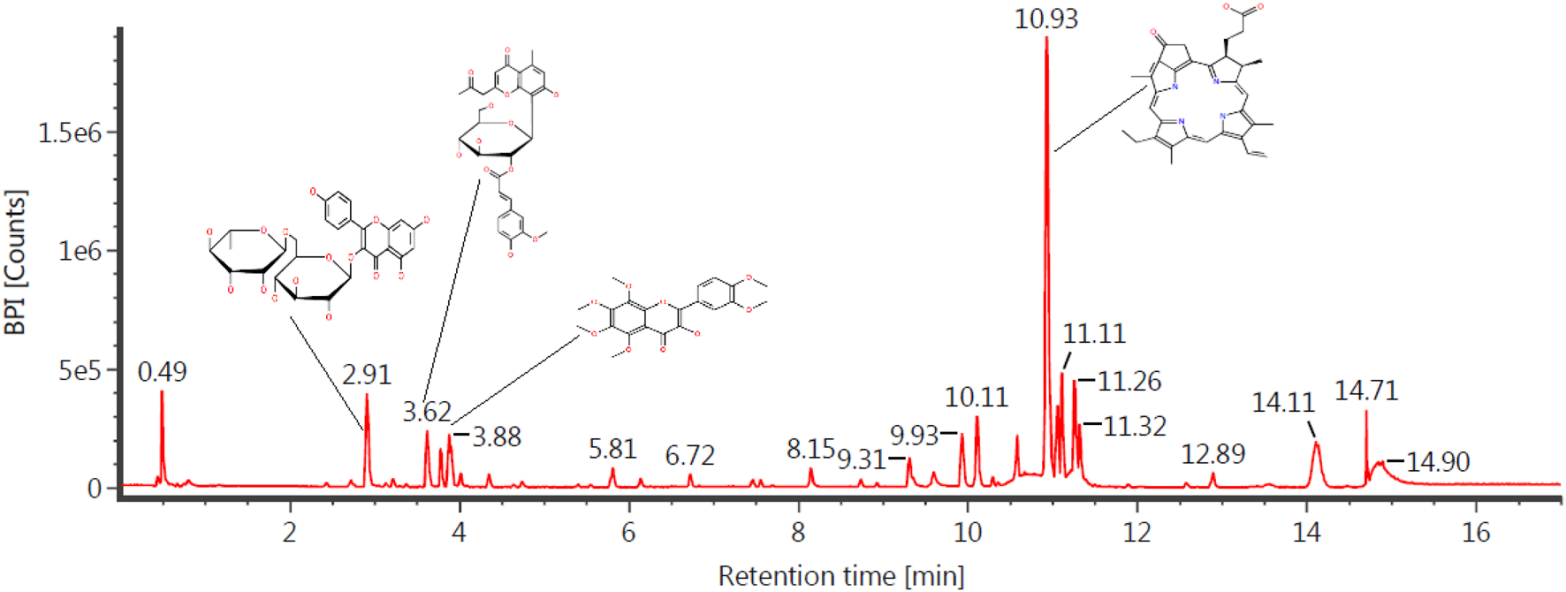
Chromatogram of *A. vera* extracts.

The presence of active substances in the functional group of *A. vera* extract is believed to have a role in inhibiting corrosion. The OH functional group in *A. vera* extract inhibits Al corrosion by 82.35% in acidic media^45^. Another study also reported that the active content in *A. vera* inhibited steel corrosion in NaCl media^40^. The dominant compound in the *A. vera* extract was identified as *methyl phaeophorbide* (10.93) and *kaempferol-3-O-rutinoside* (2.91), with the structure presented in Fig. 2. The presence of these two molecules is strongly suspected to be responsible for inhibiting corrosion.

Other compounds in the resulting *A. vera* extract are *2"-O-Feruloylaloesin* (3.62) and *Natsudaidain* (3.88). A summary of compound names, chemical formulas, and molecular weights identified in *A. vera* extracts is tabulated in Table 1.

**Table 1:** Compound names, molecular weight, formula, and retention time in *A. vera* extracts.

S/N	Compounds	Formula	Time of retention (min)	Molecular weight (m/Z)
1	*Kaempferol-3-O-rutinoside*	C_27_H_30_O_15_	2.91	594.15847
2	*2"-O-Feruloylaloesin*	C_29_H_30_O_12_	3.62	570.17373
3	*Natsudaidain*	C_21_H_22_O_9_	3.88	418.12638
4	*Methyl phaeophorbide*	C_36_H_38_N_4_O_5_	10.93	607.29245

### Electrochemical study

#### OCP analysis

**Figure 3:**
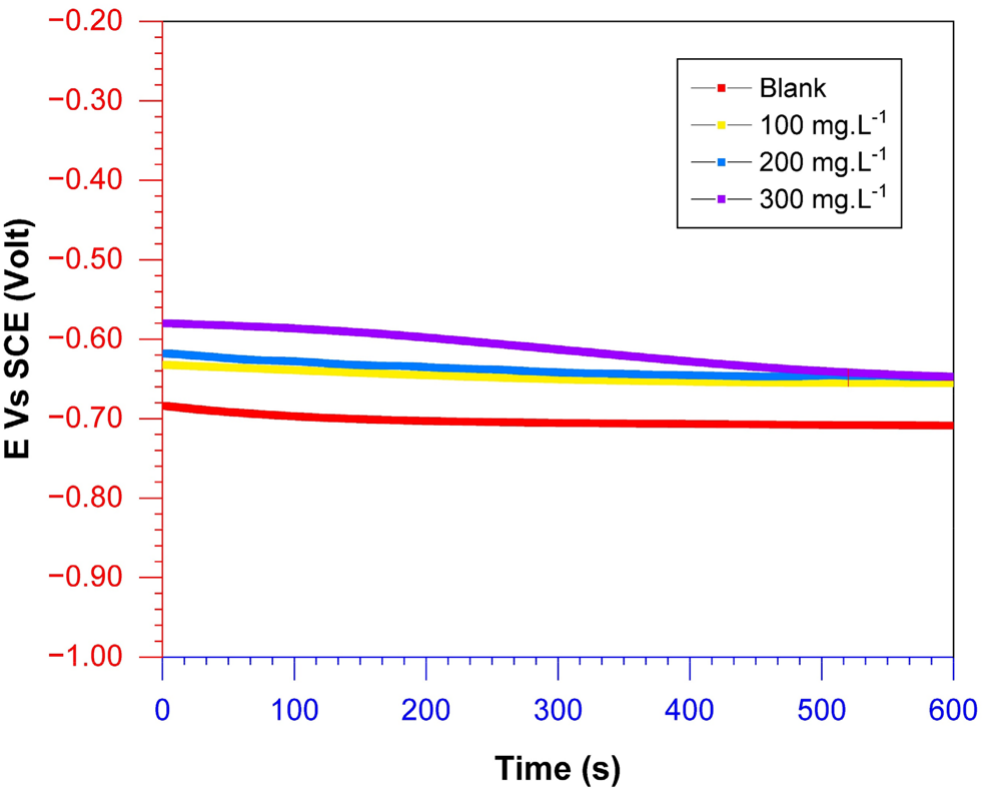
Open circuit potential (OCP) curves of API 5L steel specimens in seawater media containing various *A. vera* extracts.

Figure 3 illustrates the open circuit potential (OCP) diagram of API 5L steel specimens when exposed to synthetic seawater media, both with and without the presence of *A. vera* extract at different concentrations.

In the absence of *A. vera* extract, E_corr_ experienced initial fluctuations in the first few minutes, finally stabilizing at a value of − 0.709 V after 600 s. The observed potential decrease is consistent with an active surface, which can be associated with electrode corrosion and the formation of corrosion products that do not adequately protect the metal surface.

When exposed to *A. vera* extract, the E_corr_ value rises to greater positive levels, as shown in Table 2. The observed displacement could be ascribed to inhibiting the anodic reaction involved in iron release, as demonstrated by prior research^46,47^. The difference in corrosion potential becomes more significant as the extract concentration increases. In adding *A. vera* extract, the corrosion potential value stabilized at around -0.647 V. The observed potential shifts suggest that the inhibitory action predominantly targets anodic processes.

**Table 2:** Electrochemical open circuit potential fitting of API 5L steel in seawater media.

T (K)	C_in_ (mg L^−1^)	Crude extracts	Minimum (V vs. SCE)	Time (s)	Maximum (V vs. SCE)	Time (ms)
300	0	Blank	− 0.709	593.6	− 0.684	510.0
	100	*A. vera*	− 0.655	589.6	− 0.632	510.0
	200		− 0.648	583.6	− 0.618	510.0
	300		− 0.647	599.8	− 0.580	510.0

#### PDP analysis

**Figure 4:**
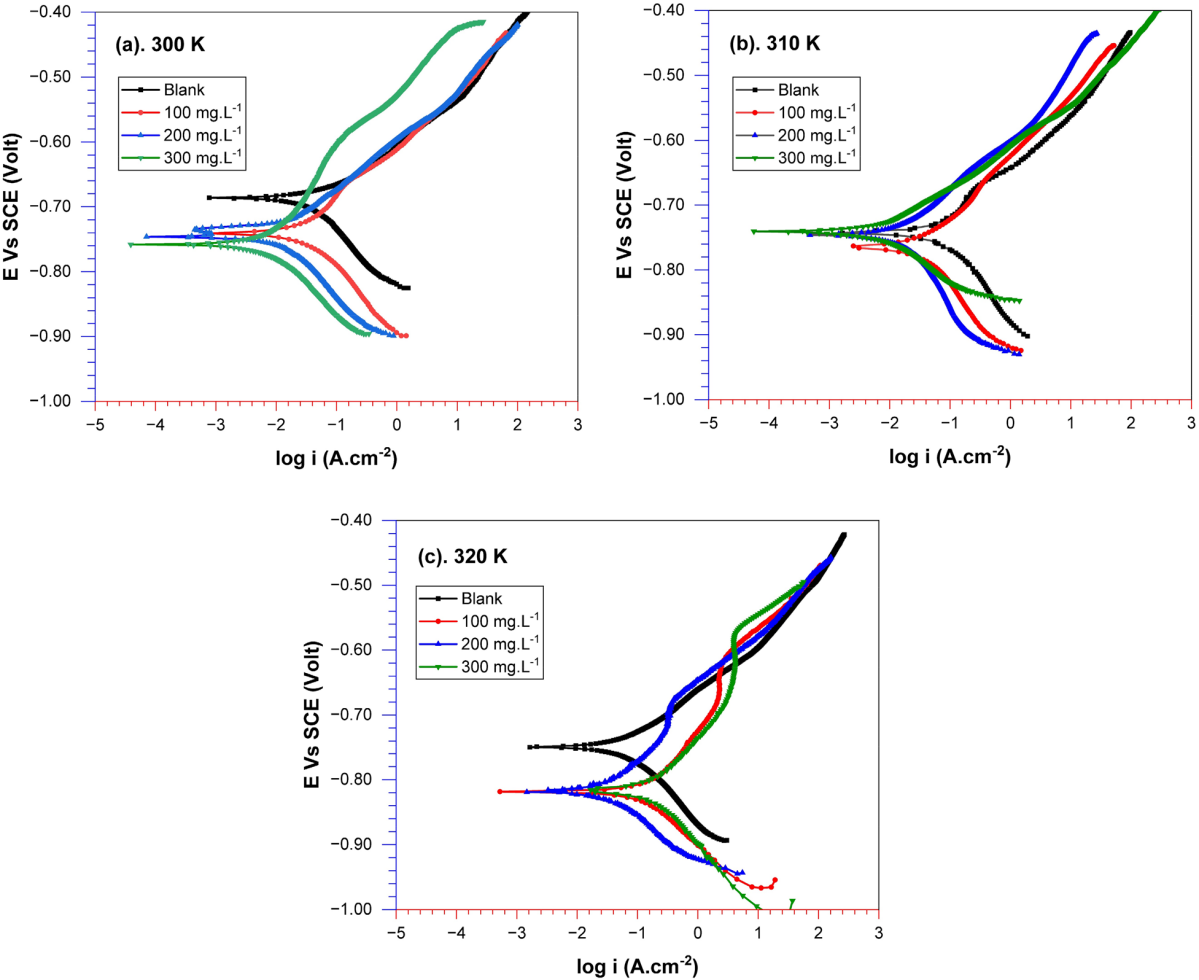
Potentiodynamic polarization curves of API 5L steel in seawater media with various *A. vera* concentrations at various temperatures.

Figure 4 illustrates the polarization curve of API 5L steel immersed in seawater media, where different concentrations of *A. vera* extract were administered at various temperatures. The potentiodynamic polarization-related data parameters are given in Table 3. It can be demonstrated that the polarization curve shows an apparent decreasing trend in the corrosion current density (i_corr_). The corrosion current density in the absence of *A. vera* is 36.48 μA cm^−2^. Meanwhile, in the presence of *A. vera* concentrations ranging from 100 to 300 mg L^−1^, the corrosion current density obtained values were very low, namely 8.25 μA cm^−2^ to 7.14 μA cm^−2^ at a temperature of 300 K. Similar behaviour was observed at temperatures of 310 and 320 K, where an increase in concentration resulted in a decrease in the corrosion current density. Electrochemical corrosion of steel surfaces in a corrosive media produces Fe^2+^ ions as an anodic reaction, followed by water dissociation at the cathodes. The presence of an inhibitor in the test solution medium resulted in a significant decrease in both the cathodic and anodic current curves, as shown in the polarisation curve (Fig. 4). This occurrence depicts the inhibitor absorbing onto the metal surface, covering the active corrosion-prone area^45^. As a result, the rate of electro-corrosive dissolution in steel is lowered. Nonetheless, the drop in cathodic current is substantially more significant than the reduction in anodic current; therefore, the inhibitor's cathodic features take precedence.

**Table 3:** Electrochemical potentiodynamic polarization results of API 5L steel in seawater medium with various *A. vera* concentrations for different temperatures.

T (K)	C_in_ (mg L^−1^)	−β_c_ (mV/dec)	E_corr_ (mV)	i_corr_ (μA/cm^2^)	Corr. rate (mpy)	θ	IE (%)
300	0	110.1	− 685.70	36.48	16.67	0.00	0.00
	100	75.14	− 740.40	8.247	3.77	0.77	77.40
	200	66.84	− 745.40	7.713	3.52	0.79	78.86
	300	95.12	− 756.00	7.138	3.26	0.80	80.43
310	0	124.90	− 740.80	44.15	20.17	0.00	0.00
	100	89.38	− 763.00	24.5	11.19	0.45	44.52
	200	118.10	− 744.30	15.09	6.89	0.66	65.82
	300	78.04	− 743.00	7.173	3.28	0.84	83.75
320	0	82.77	− 749.60	55.65	25.43	0.00	0.00
	100	52.02	− 818.40	27.33	12.49	0.51	50.88
	200	49.55	− 819.00	19.99	9.14	0.64	64.08
	300	77.42	− 815.70	12.07	5.52	0.78	78.31

In the presence of inhibitors, the corrosion potential of API 5L (E_corr_) steel tends to shift towards the cathodic side when it is immersed in artificial seawater. The observed behaviour indicates that the *A. vera* adsorption on the investigated samples hinders the dissolution of API 5L steel and restricts the movement of reactants between the bulk solution and the metal surface^45^. However, the E_corr_ shift tends towards the anodic area along with the addition of *A. vera* extract, so it did not characterize the inhibitor's behavior as pure cathodic type and indicated a mixed type of environmentally corrosion inhibitor. Therefore, it can be said that *A. vera* extract functions as a corrosion inhibitor, which shows mixed corrosion inhibition for API 5L steel in various media, as evidenced by studies^37,38,41,45^.

Furthermore, based on several previous literature reports, if the difference in _corr_ corrosion potential (E_corr_) between the absence and presence of a corrosion inhibitor is less than 85 mV, then the inhibitor could be classified as a mixed-type inhibitor^40,41,48^. Otherwise, it will be categorized as either anodic or cathodic, as stated in reference^49,50^. The data in Table 3 and the plot in Fig. 4 indicate that the corrosion potential (E_corr_) change is less than 85 mV towards the cathodic area. This data suggests that *A. vera* is of a mixed type, predisposed towards cathodic behavior at low frequencies and anodic behavior at high frequencies. In other words, *A. vera* preferentially adsorbs on cathodic sites and greatly influences the dissolution of mild steel at low temperatures. Similarly, in another study, *A. vera* extract in a 3% NaCl medium was classified as a mixed-type corrosion inhibitor with cathodic tendencies^51^.

Table 3 also shows that as the concentration of the inhibitor studied increases, the i_corr_ value decreases, but the inhibition efficiency (IE) increases. However, with increasing temperature, corrosion inhibition efficiency tends to decrease. This case demonstrates that *A. vera* extract can successfully decrease API 5L steel corrosion in marine environments at room (300 K). This situation is indicated by the inhibition efficiency value, which tends to rise with increasing inhibitor concentration and decline with rising medium temperature. The efficiency value (IE) of the *A. vera* inhibitor for API 5L steel is determined from corrosion current density (i_corr_) data by referring to Eq. (1). The maximum inhibitory power reaching 83.75% was obtained by adding 300 mg L^−1^ of *A. vera* extract at a temperature of 310 K. A study on steel in an atmosphere containing hydrochloric acid also revealed this correlation, with the effectiveness of reducing the rate of corrosion of steel decreasing somewhat as temperature increased and being in line with inhibitor concentration^52^. The influence of *A. vera* concentration on bronze was also investigated in chloride media, and it was discovered that its inhibitory effectiveness increased with raising inhibitor, reaching 86% at 750 ppm^36^. This inhibition is related to the adsorption of active substance electrons, thus blocking the active area of API 5L steel.

#### EIS analysis

**Figure 5:**
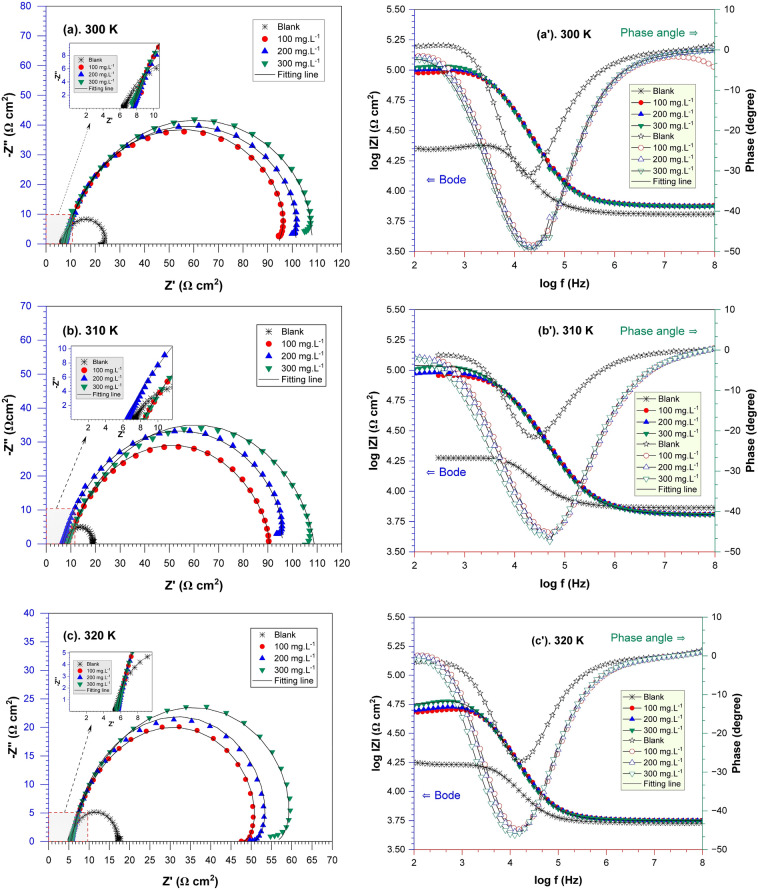
Nyquist and Bode plots of API 5L steel in seawater media with various *A. vera* concentrations at different temperatures.

Nyquist and Bode's plots illustrate the behavior of API 5L steel in seawater media with various concentrations of *A. vera* extract at different temperatures, as presented in Fig. 5.

Figures 5a–c display the Nyquist plots acquired for API 5L steel submerged in seawater media, both in the absence and presence of different inhibitor concentrations. The figure illustrates that the Nyquist plot is characterized by slightly depressed semicircles, suggesting that corrosion in API 5L steel primarily happens through a charge transfer mechanism^53^. Another study stated that capacitive arc reactance in mild steel immersed in solution is influenced by charge transfer mechanisms at the interface between the solution and surface metal^1^. Surface inhomogeneities or interface phenomena can contribute to forming curve deviations from the ideal semicircle^26^. In addition, imperfect semicircular capacitive loops can be attributed to dispersion effects caused by the non-uniformity of the metal surface^54^. Figure 5 shows that the diameter of the semicircle expands when the *A. vera* extracts are added to the synthetic seawater solution. This enlargement of the semicircle continues as the inhibitor concentration rises. This result clarifies why API 5L steel surfaces develop an inhibitor layer that prevents corrosion.

**Figure 6:**
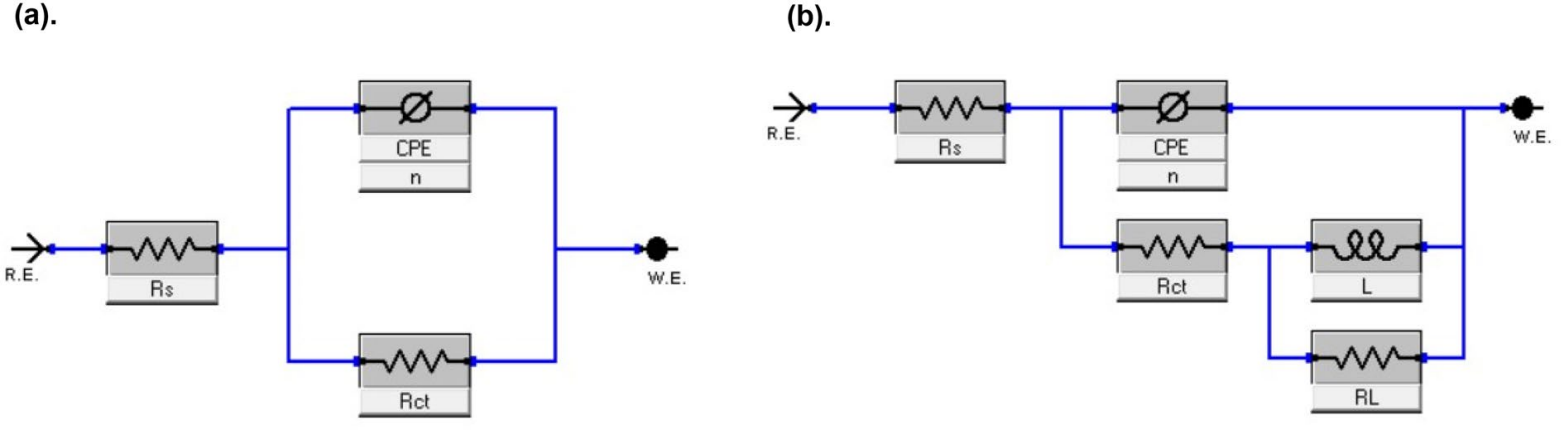
Model of circuit used for *A. vera* extract performance.

The impedance spectrum of the blank medium displays a modest and incomplete inductive impedance induced by the adsorption–desorption process of the inactive active ingredient on the metal surface. Figure 6a shows the analogous circuit for this circumstance. Another electrical circuit is supplied for the situation by including an inhibitor (Fig. 6b) to produce a better simulation of the low-frequency inductance loop, which has an inductor resistance (R_L_) and is in series with the inductor (L)^55^. The relaxation of inhibitors causes the inductive loop seen on the Nyquist plot adsorbed on the metal surface^56^. Due to corrosion-induced surface heterogeneity, the Nyquist plot displays a depressed semicircle rather than an ideal semicircle. To generate an excellent relationship between experimental impedance data and simulated, CPE was utilized instead of ideal double-layer capacitance in the circuit. Singh et al. define CPE impedance as the following equation^57^:

3 $${Z}_{CPE}={Y}_{0}^{-1}{\left(j\omega \right)}^{-n}$$

Y_0_ is the quantity of CPE (constant phase element), j^2^ = − 1 is an imaginary integer, ω denotes the angular frequency, and n represents the exponent. The value of n, where − 1 ≥ n ≥ 1, indicates the characteristics of the CPE, which were described in the previous study^57,58^. The double layer capacitance (C_dl_) value is determined using the formula Eq. (4), shown by the CPE installation results.

4 $${C}_{dl}={Y}_{0}{\left({\omega }_{max}\right)}^{n-1}$$

The efficiency of *A. vera* extracts was estimated from R_ct_ data using Eq. (2)^44^, and the installed impedance parameters are presented in Table 4. These parameters include charge transfer resistance (R_ct_), solution resistance (R_s_), and corrosion inhibition efficiency (IE%). Calculations for R_ct_, inductive reactance (L), and their associated resistance (R_L_), as well as the results of installing constant phase elements (CPE), are used to determine these values. The impedance associated with a Constant Phase Element (CPE) can be represented mathematically, as shown in reference^57^.

**Table 4:** Electrochemical impedance fitting results of API 5L steel in seawater medium with various *A. vera* concentrations for different temperatures.

T (K)	C_in_ (mg L^−1^)	R_s_ (Ω cm^2^)	R_ct_ (Ω cm^2^)	Q_dl_ × 10^–6^ (S*s^a^)	n	R_L_ (Ωcm^2^)	L (H cm^2^)	Error	θ	IE (%)
300	0	6.54	17.97	1002.0	0.95	–	–	0.00188	0.00	0.00
	100	7.66	87.51	541.5	0.86	6.50	2.112	0.00240	0.79	79.47
	200	7.55	94.61	618.6	0.84	8.32	2.345	0.00014	0.81	81.01
	300	7.51	101.40	643.9	0.84	9.20	2.458	0.00019	0.82	82.28
310	0	7.37	11.90	1480.0	0.87	–	–	0.00029	0.00	0.00
	100	6.49	84.12	482.4	0.79	3.96	1.551	0.00037	0.86	85.85
	200	6.45	90.25	539.4	0.77	5.59	1.790	0.00011	0.87	86.81
	300	6.44	104.40	589.2	0.77	5.69	1.955	0.00028	0.89	88.60
320	0	5.39	12.03	2150.0	0.90	–	–	0.00049	0.00	0.00
	100	5.65	42.60	1299.0	0.85	7.89	2.156	0.00021	0.72	71.76
	200	5.68	45.30	1115.0	0.88	8.75	2.371	0.00107	0.73	73.44
	300	5.61	51.27	1492.0	0.84	9.93	3.784	0.00010	0.77	76.54

Bode plots are illustrated in Fig. 5aʹ–cʹ. The Bode plots demonstrate that increasing the concentration of *A. vera* extract results in an increase in impedance (|Z|) at low frequencies, which implies good corrosion inhibition efficacy^59^. Moreover, the curve of the Bode phase plot indicates that the phase angle increases with increasing *A. vera* concentration, reaching a maximum of − 49.42^0^, − 47.39^0^, and − 46.28^0^ with the addition of 300 mg L^−1^ extracts at 300, 310, and 320 K, respectively. This results in more significant inhibitor adsorption, which is related to improved capacitive performance^57^.

Additionally, a comparison was made between the corrosion ability of *A. vera* and other plant extracts in the existing literature on the behavior of steel in seawater medium. According to the study, the *A. vera* extracts could reduce steel corrosion by 82.28% when adding 300 mg L^−1^ of concentration at 300 K of media temperature. In comparison, chamomile flower extract had an inhibitory efficiency of 75.66% at 20 mL/L in artificial seawater^60^, while *Acacia Tortilis* bark extract showed a slightly lower efficiency, namely 72.9%^15^. Moreover, the maximum iron inhibition efficiency was 86.4%, with a high concentration (3000 ppm) for Terebinth extracts in 3% NaCl media at room temperature^19^. These findings indicate that *A. vera* is a highly effective corrosion inhibitor.

### Adsorption, thermodynamics, and kinetics analysis

**Figure 7:**
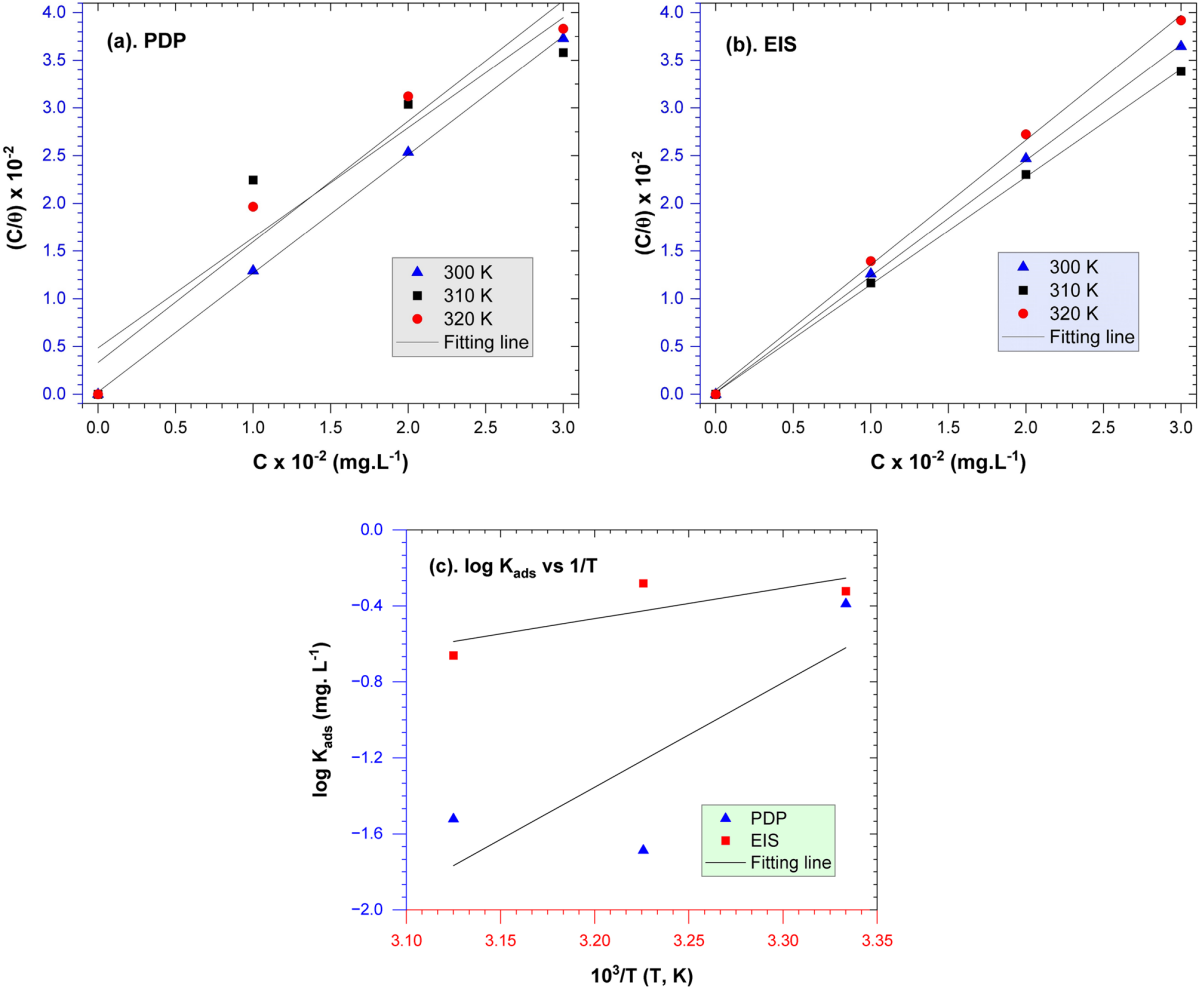
Langmuir isotherm of the adsorption for (**a**) PDP data, (**b**) EIS data, and (**c**) log K_ads_ plots versus solution temperature.

To study the mechanism of corrosion inhibition of *A. vera* on API 5L steel in seawater media, the adsorption properties of *A. vera* on the steel surface and thermodynamics were analyzed using the information data presented in Tables 3 (PDP) and 4 (EIS). The experimental data show good agreement with the Langmuir isotherm equation (Eq. 5), as reported in the previous work^7^.

5 $$\frac{C}{\theta }=\frac{1}{{K}_{ads}}+C$$

where C symbolizes the concentration of *A. vera* extract and is a closed surface taken from PDP and EIS measurements, the results of the fitting process between C/θ versus C for each PDP and EIS data are depicted in Fig. 7a and b. The equilibrium of the adsorption constant (K_ads_) value was determined and presented in Table 5 based on the intersection of the fitted straight lines. The value of K_ads_ showed a negative relationship with temperature, confirming that the adsorption efficiency of *A. vera* decreases as the temperature increases. The free energy of adsorption (ΔG_ads_) value for *A. vera* extracts on the API 5L steel surface was determined according to Eq. (6), and the adsorption enthalpy value (ΔH_ads_) was calculated using Eq. (7) as explained in reference^61^. The ΔG_ads_, ΔH_ads_, and ΔS_ads_ values were obtained from calculations presented in Table 5. Figure 7c presents a graphical representation of the fitting results for the logarithmic function of K_ads_ and the inverse of temperature (1000/T). The data analysis reveals that the adsorption of *A. vera* active substances on the steel surface tends to be a physisorption-chemisorption and exothermic process (negative ΔH_ads_ value).

**Table 5:** Thermodynamics parameters of *A. vera* extracts in seawater media at different temperatures.

T (K)	PDP					EIS				
	R^2^	K/(mg L^−1^)	∆G (kJ mol^−1^)	∆H (kJ mol^−1^)	∆S (kJ mol^−1^ K^−1^)	R^2^	k/(mg L^−1^)	∆G (kJ mol^−1^)	∆H (kJ mol^−1^)	∆S (kJ mol^−1^ K^−1^)
300	0.9997	0.40816	− 32.229	− 105.445	− 105.338	0.9998	0.47619	− 32.614	− 30.697	− 30.588
310	0.8931	0.02058	− 25.603		− 105.363	0.9997	0.52356	− 33.946		− 30.587
320	0.9523	0.03012	− 27.442		− 105.359	0.9988	0.21834	− 32.713		− 30.594

6 $${\Delta G}_{ads}^{0}=-2.303\,RT\,log\left({10}^{6}\,{K}_{ads}\right)$$

7 $${\Delta G}_{ads}^{0}={\Delta H}_{ads}^{0}-T{\Delta S}_{ads}^{0}$$

Temperature is a crucial variable that must be evaluated in addition to the adsorption process to understand thermodynamic phenomena and reaction kinetics. A significant increase in temperature can affect various material attributes such as corrosion rate, equilibrium constant, and kinetics. The Arrhenius equation (Eq. 8) could be used to study the correlation between temperature factors and corrosion rate, as shown as follows^62^:

8 $$\text{log}CR=-\left(\frac{{E}_{a}}{2.303R}\right)\frac{1}{T}+\text{log}A$$

**Figure 8:**
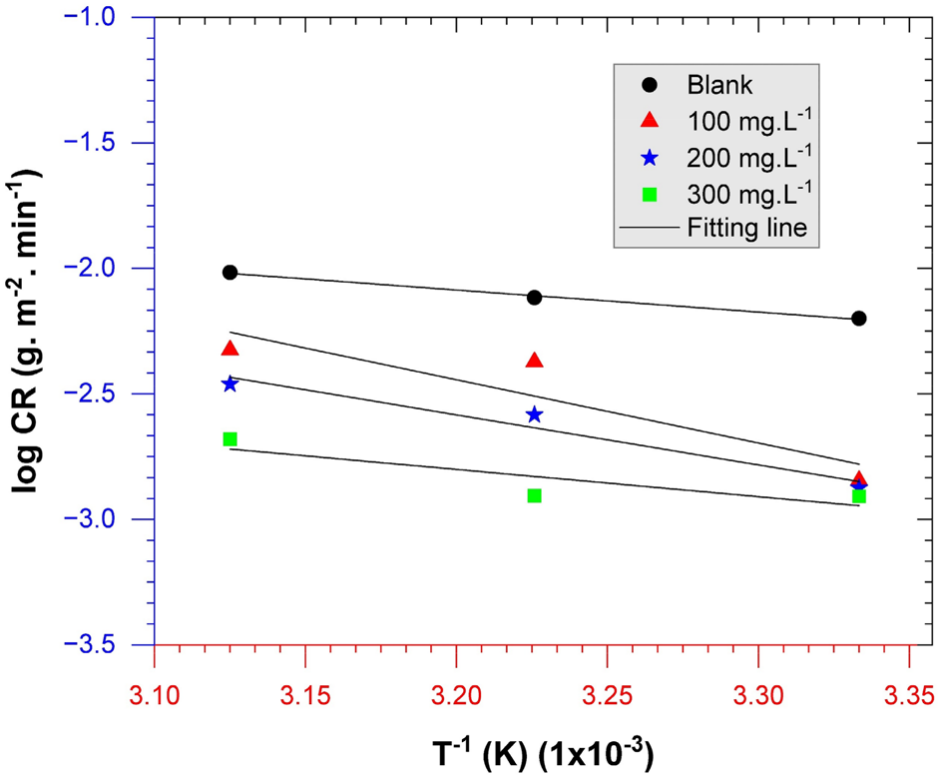
Arrhenius plots of log CR versus temperature.

CR represents the corrosion rate, E_a_ denotes the activation energy, R is the gas constant (J.K^−1^ mol^−1^), T is the absolute temperature (K), and A is the pre-exponential constant. The activation energy of the reaction could be calculated from the plot of log CR versus 1/T, as illustrated in Fig. 8. The activation energies to every extract concentration as an inhibitor are 0 mg L^−1^, 100 mg L^−1^, 200 mg L^−1^, and 300 mg L^−1^ are 16.832, 20.738, 38.174, and 48.235 kJ.mol^−1^, respectively. A summary of activation energy parameter data at various inhibitor concentrations is tabulated in Table 6. The activation energy is more significant with the addition of *A. vera* extract than without the addition of the extract because of the adsorption process^53^. The rising E_a_ with increasing temperature indicates the bioactive molecules' adsorption process on the steel surface^63^. Based on several studies of plant extracts, adding extracts could drastically alter the morphology of surface API 5L steel, the chemical characteristics, and surface roughness following exposure^64^.

**Table 6:** Energy activation parameters of *A. vera* inhibition.

C_in_ (mg L^−1^)	R^2^	E_a_ (kJ mol^−1^)	A (g m^−2^ min^−1)^	log A
0	0.9944	16.832	5.34E+00	0.7274
100	0.7408	20.738	4.63E+00	0.6655
200	0.9552	38.174	6.26E+03	3.7966
300	0.8323	48.235	4.15E+05	5.6182

### Effect of temperature and time

**Figure 9:**
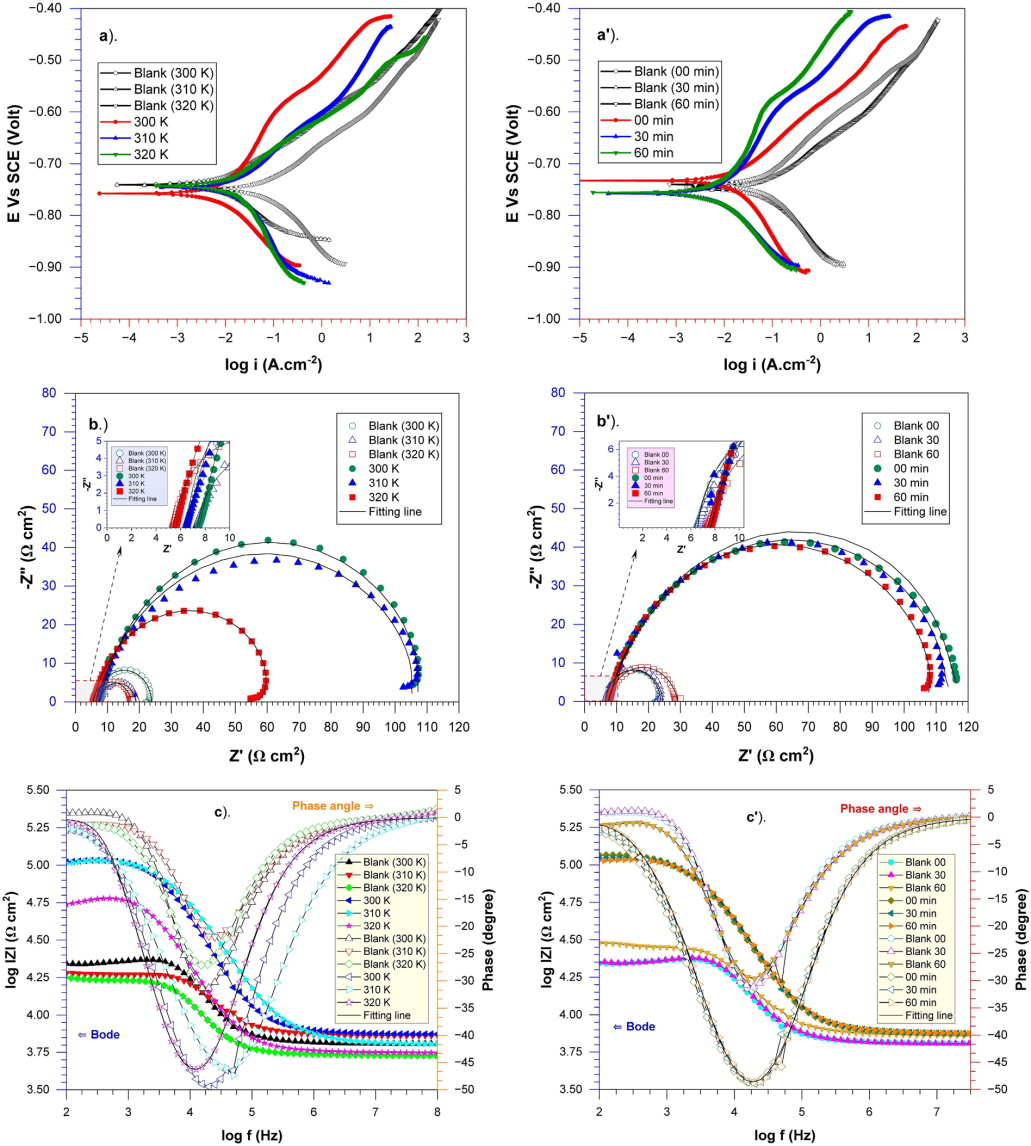
The temperature and immersion time effects on API 5L steel in seawater media containing 300 mg L^−1^ of *A. vera* extracts for (**a**,**aʹ**) PDP, (**b**,**bʹ**) Nyquist, and (**c**,**cʹ**) Bode curves.

The inhibitor performance was evaluated based on media temperature and immersion time to provide an overview of the optimal temperature and injection time in inhibitor applications. The evaluation results of API 5L steel based on solution temperature and immersion time with 300 mg L^−1^ *A. vera* extract are presented in Fig. 9. Electrochemical parameter data resulting from the influence of temperature and time are tabulated in Table 7 and Table 8.

**Table 7:** The temperature and immersion time affect PDP parameters for API 5L steel in seawater media containing 300 mg L^−1^ of *A. vera* extracts.

Temp. (K)	Time (min)	C_in_ (mg L^−1^)	−β_c_ (mV dec^−1^)	E_corr_ (mV)	i_corr_ (μA cm^−2^)	Corr. rate (mpy)	IE (%)
Blanko	30	0	100.20	− 741.70	36.64	16.64	0.00
300		300	100.40	− 756.00	7.72	3.53	78.82
Blank		0	71.65	− 740.80	46.04	21.04	0.00
310		300	100.10	− 744.30	12.74	5.82	72.32
Blank		0	84.70	− 479.60	57.34	26.20	0.00
320		300	155.80	− 742.10	16.80	7.68	70.71
300	Blank	0	59.22	− 744.50	39.55	18.07	0.00
	0	300	60.15	− 732.50	7.50	3.43	81.03
	Blank	0	59.39	− 749.60	36.79	16.81	0.00
	30	300	95.80	− 756.00	7.21	3.30	80.39
	Blank	0	63.47	− 740.00	35.85	16.38	0.00
	60	300	106.80	− 752.60	7.17	3.28	79.99

**Table 8:** The temperature and immersion time affect EIS parameters for API 5L steel in seawater media containing 300 mg L^−1^ of *A. vera* extracts.

Temp. (K)	Time (min)	C_in_ (mg L^−1^)	R_s_ (Ω cm^2^)	R_ct_ (Ω cm^2^)	Q_dl_ × 10^–6^ (S*s^a^)	n	R_L_ (Ω cm^2^)	L (H cm^2^)	Error	IE (%)
Blank	30	0	6.55	17.29	998.9	0.97	–	–	0.00140	0.00
300		300	7.51	100.40	643.9	0.84	9.93	3.784	0.00019	82.78
Blank		0	7.36	11.63	1387.0	0.89	–	–	0.00028	0.00
310		300	6.44	99.74	589.2	0.80	6.50	2.712	0.00028	88.34
Blank		0	5.39	12.03	2153.0	0.90	–	–	0.00049	0.00
320		300	5.61	51.67	1492.0	0.84	6.50	2.695	0.00010	76.72
300	Blank	0	6.55	17.29	998.9	0.96	–	–	0.00140	0.00
	0	300	7.50	110.70	811.2	0.81	8.97	2.237	0.00019	84.38
	Blank	0	6.54	17.96	1002.0	0.95	–	–	0.00188	0.00
	30	300	7.59	107.70	694.7	0.81	7.16	2.139	0.01691	83.32
	Blank	0	7.35	21.80	1287.0	0.87	/	/	0.00069	0.00
	60	300	7.55	100.60	693.6	0.83	5.90	3.887	0.00009	78.33

Potentiodynamic polarization and impedance data based on temperature show that with increasing solution temperature, the inhibition efficiency (IE) value decreases. The optimum efficiency value obtained was 78.82% for PDP and 88.34% for EIS. This phenomenon of lower efficiency can be explained by the fact that green inhibitors from plant extracts are readily degraded or decomposed at high temperatures^50^. This finding aligns with previous studies on *A. vera* extracts as a corrosion inhibitor for mild steel in a 15% HCl, where inhibition efficiency was affected by temperature^65^. Another study states that the efficiency of *A. vera* extracts reduced at high media temperatures was caused by internal competition in the adsorption and desorption forces of specific inhibitor molecules involved in the corrosion inhibition at active areas on the steel surface^66^. Similar results were also found in other extracts where the inhibition efficiency decreased at higher temperatures^17,27^.

A decrease in efficiency is also seen when the soaking time is extended in both PDP and EIS results. This phenomenon of decreased value is caused by adsorption on the metal surface, and then the concentration decreases with the length of soaking time. As immersion time rises, the corrosion potential slightly moves in the cathodic direction (Fig. 9a), and the diameter of the semicircle of the Nyquist curve increases (Fig. 9b). R_ct_ will decrease over time due to a decrease in the stability of the protective layer because of the diffusion and desorption of bioactive extract molecules toward the electrolyte interface or protective layer. Moreover, in the Bode phase plots, the phase angle decreases with increasing temperature and immersion time (Fig. 9c and cʹ). Soaking times have a significant effect on corrosion phenomena on metal surfaces, and consequently, several studies have evaluated the performance of inhibitors based on exposure time^12,19,40,48^.

Other studies have evaluated the effects of temperature and time, such as the study on the performance of extracts obtained from ginger and methanol as a solvent^67^. According to their investigation, 200 mg L^−1^ at 298.15 K enhanced inhibition efficiency by up to 94%. Regia fruit peel extract in the 3.5% sodium chloride solutions was also studied as an organic corrosion inhibitor for mild steel and revealed that the inhibitory efficiency increased with soaking times over 48 h^16^. The optimal efficiency was achieved at around 94% by adding 1000 mg L^−1^ extract. Another study evaluated the corrosion inhibition efficiency of imidazoline on Q235 steel in artificial seawater containing 3.5% NaCl as a corrosive media^68^. The efficiency of inhibition was increased with the increase in temperature and concentration of extract. The optimum corrosion inhibition performance was obtained at approximately 90.69% for the PDP method and 95.95% for the EIS method, respectively^68^.

Furthermore, the performance study of *A. vera* on low-carbon steel in acid environments (HCl and HNO_3_) revealed that inhibitory efficiency decreased as temperature and soaking duration increased. The study discovered that *A. vera* extracts could act as inhibitors, with efficiency of up to 77.32%^40^. The investigation of *A. vera* at different times (in 3-day intervals) in H_2_SO_4_ solution also showed that the weight loss of mild steel decreased as the activity of *A. vera* decreased over time^48^.

### Comparative efficiency and future study prospects

A careful comparative analysis was carried out to provide a comprehensive evaluation of the performance of *A. vera* as a green inhibitor, as tabulated in Table 9. This analysis was carried out to contextualize the performance of *A. vera* and highlight the potential of plant extracts as a new and promising biomaterial in inhibiting steel corrosion. Table 9 compares this study's results with several recent studies in the development of *A. vera* extracts as green corrosion inhibitors (last ten years). In this study, the corrosion rate inhibitor efficiency (IE) was around 83.75% (PDP) and 88.60% (EIS). The findings of this study show extraordinary corrosive inhibition performance, with only a relatively small dose addition (300 mg L^−1^) able to inhibit steel corrosion in a seawater environment above 80%. However, the efficiency value in this study is still relatively small compared to some literature (Table 9). Therefore, extra studies are needed, such as extracting and isolating bioactive ingredients that prevent corrosion, to open future research avenues for developing corrosion inhibitors that are environmentally friendly, efficient, cost-effective, and sustainable in the marine environment.

**Table 9:** Comparative efficiency of *A. vera* extracts with several previous studies.

S/N	Inhibitor study (years)	Metal/medium	Inhibitor concentration	Inhibitor efficiency	Ref
1	*Aloe vera* (2015)	Iron metal sheets/1 M HCl	200 mg kg^−1^	83.59%	^69^
2	*Aloe vera* gel (2016)	Mild steel/1 M HCl	200 ppm	87.4%	^57^
3	*Aloe vera* gel (2017)	Mild steel/0.5 M CH_3_COOH	1.0 g L^−1^	82.35%	^45^
4	*Aloe vera* (2018)	Mild steel/0.1–0.5 M HCl-HNO_3_	0.03 g L^−1^	77.32%	^40^
5	*Aloe vera* (2019)	Aluminium alloys/3% NaCl	0.08 mg mL^−1^	93.25%	^39^
6	*Aloe vera* (2020)	Mild steel/0.5 M H_2_SO_4_	10% (vol.)	88.9%	^48^
7	*Aloe polysaccharide (APS)* (2020)	Mild steel/15% HCl	800 ppm	96.61%	^59^
8	*Aloe vera* gel (2021)	Carbon steel/1.0 M HCl	100 ppm	52%	^38^
9	*Aloe vera* (2022)	Medium carbon steel/1 M H_2_SO_4_	800 ppm	> 90%	^37^
10	*Aloe vera* (2022)	Medium carbon steel (0.39% C)/seawater	4%	85%	^70^
11	*Aloe saponaria* (2023)	Bronze B66/neutral chloride (3% NaCl)	750 ppm	88%	^71^
12	*Aloe vera* gel (2024)	Reinforcement steel/0.5 M NaCl	20%	86%	^72^
13	*Aloe ferox mill* (2024)	Copper/1.0 M HCl	250 mg L^−1^	93.3%	^73^
14	*Aloe vera barbadensis* (2024)	Mild steel/1 M H_2_SO_4_	1.5 g L^−1^	77%	^74^
15	*Aloe vera (L.) Burm. f* (2024)	API 5L steel/synthetic seawater	300 mg L^−1^	83.75% (PDP) and 88.60% (EIS)	In this work

### Surface analysis

#### SEM/EDS analysis

**Figure 10:**
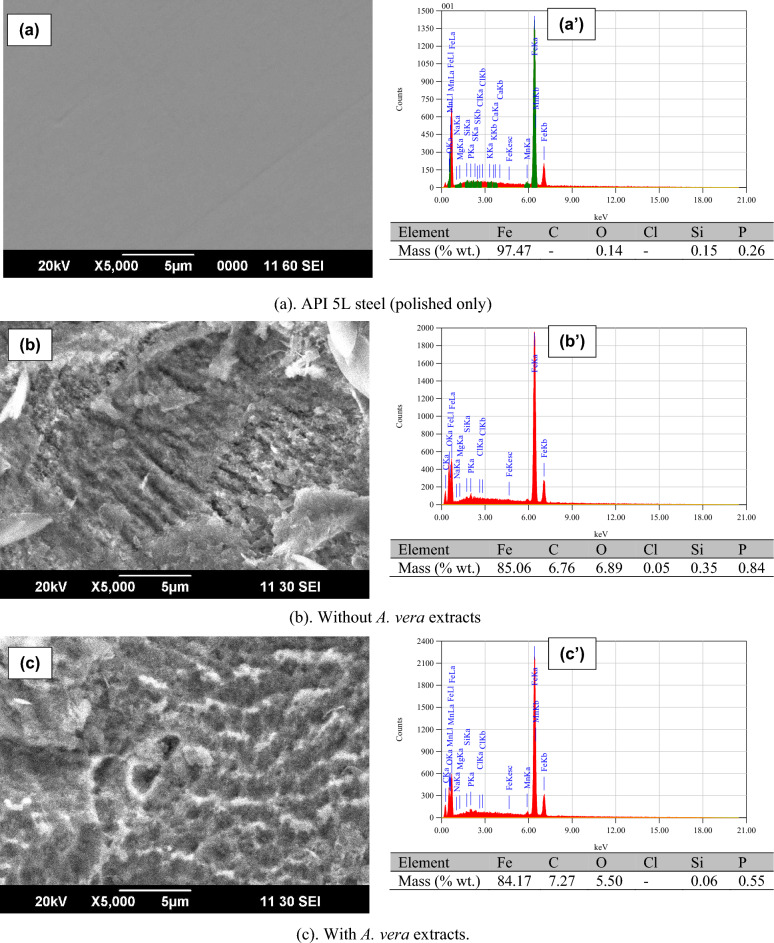
SEM/EDS results on the API 5L steel surface after immersion (72 h) in a seawater environment.

After the experiment, the surface corrosion morphology of the API 5L steel samples was analyzed using scanning electron microscopy (SEM). For this analysis, the API 5L steel was immersed for 72 h. The results obtained are presented in Fig. 10. Morphological results show that the solution without the extract has caused significant surface damage to API 5L steel due to corrosion. On the contrary, the surface of API 5L steel remains smooth in the solution inhibited by the extract, indicating that the use of *A. vera* extract significantly inhibits the corrosion reaction of API 5L steel in an artificial seawater environment.

In solution without the extract, Fig. 10 also shows the elemental composition of the metal surface, which mainly consists of Fe and O. The presence of Fe and O indicates the formation of iron oxide on the surface of the metal. The decrease of O element took place in synthetic seawater by adding the extract. This decrease implies that there is a less dense corrosion product, such as iron oxide, when adding the extract.

#### AFM analysis

**Figure 11:**
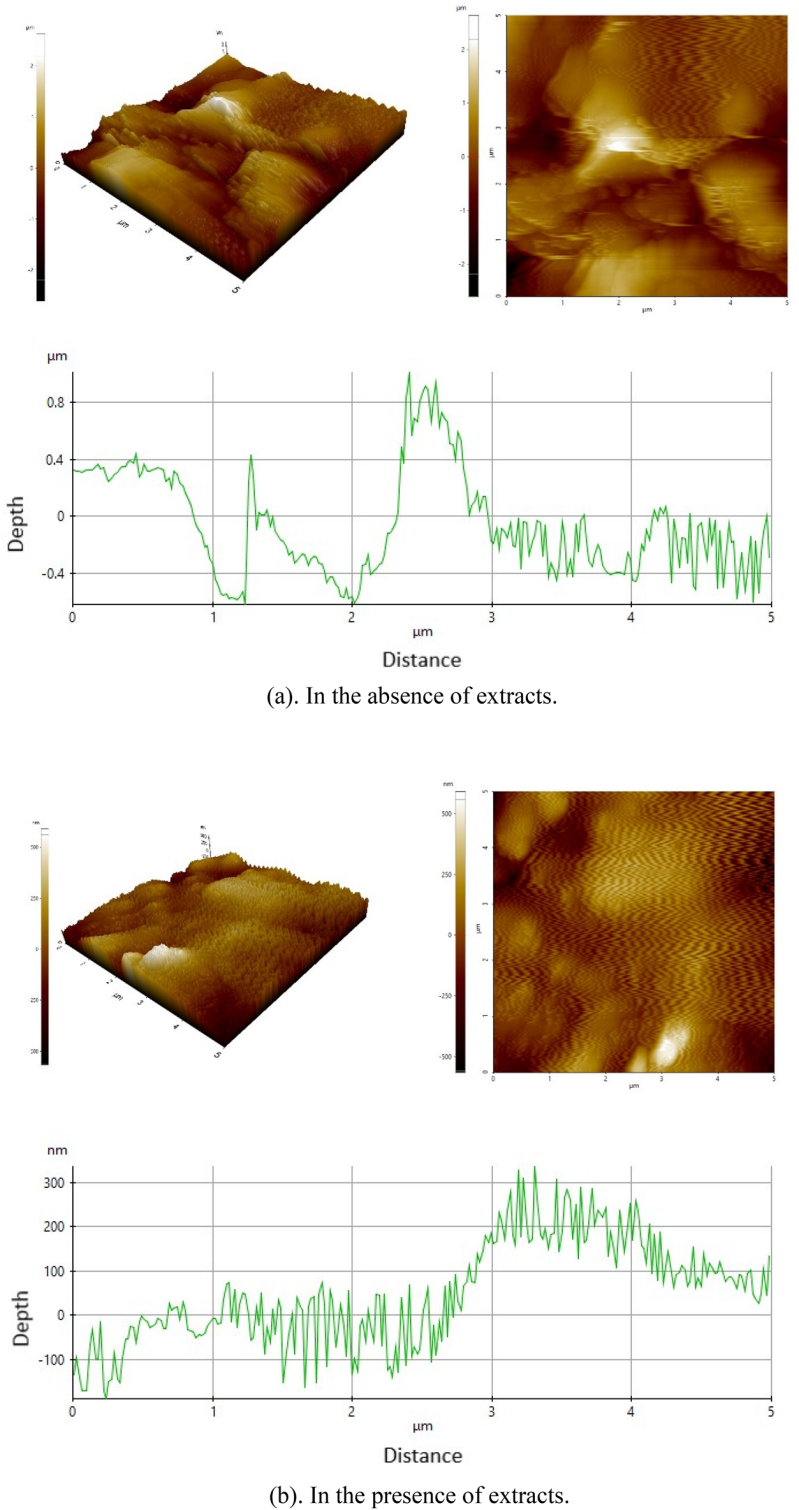
3D, 2D AFM morphology profile of steel surface.

AFM data describe steel's surface morphology and inhibitors' effect on the formation and development of corrosion at the interface between the metal and solution^27^. Three-dimensional (3D) and two-dimensional (2D) AFM morphology profiles for API 5L steel surfaces without and with the presence of *A. vera* extract soaked in seawater media are shown in Fig. 11, respectively.

In addition to surface morphology, nominal quantitative mean roughness deviation (R_a_) and root mean square roughness (R_q_) of a metal surface can be obtained from AFM analysis. The R_a_ and R_q_ values for the steel surface without any extract (blank specimen) are 0.424 µm and 0.5579 µm, respectively. The visible roughness on the steel surface without extract is caused by corrosion attack and the absence of surface protection. This data shows that the surface of API 5L steel exposed in a seawater environment without inhibitors (extract) has a rougher surface morphology than the metal surface treated with the inhibitor, indicating that the API 5L steel surface is not protected, resulting in a rougher morphology, as seen in Fig. 11a. In contrast, Fig. 11b shows a smoother surface in seawater media containing 300 mg L^−1^ *A. vera* extract. The surface smoothness is generated by forming a compact protective layer of Fe^2+^ and the Fe(OH)_2_ complex on the metal surface because of the interaction of the extract's active components, which inhibits corrosion on surface steel^75^. The average R_a_ and R_q_ values of API 5L steel surfaces are 0.1104 µm and 0.1429 µm, respectively. The R_a_ and R_q_ values were significantly lower in the extract-impeded environment than in the unconstrained environment. This parameter ensured that the surface was smoother than the steel surface without inhibitors.

### Mechanism of corrosion inhibition

**Figure 12:**
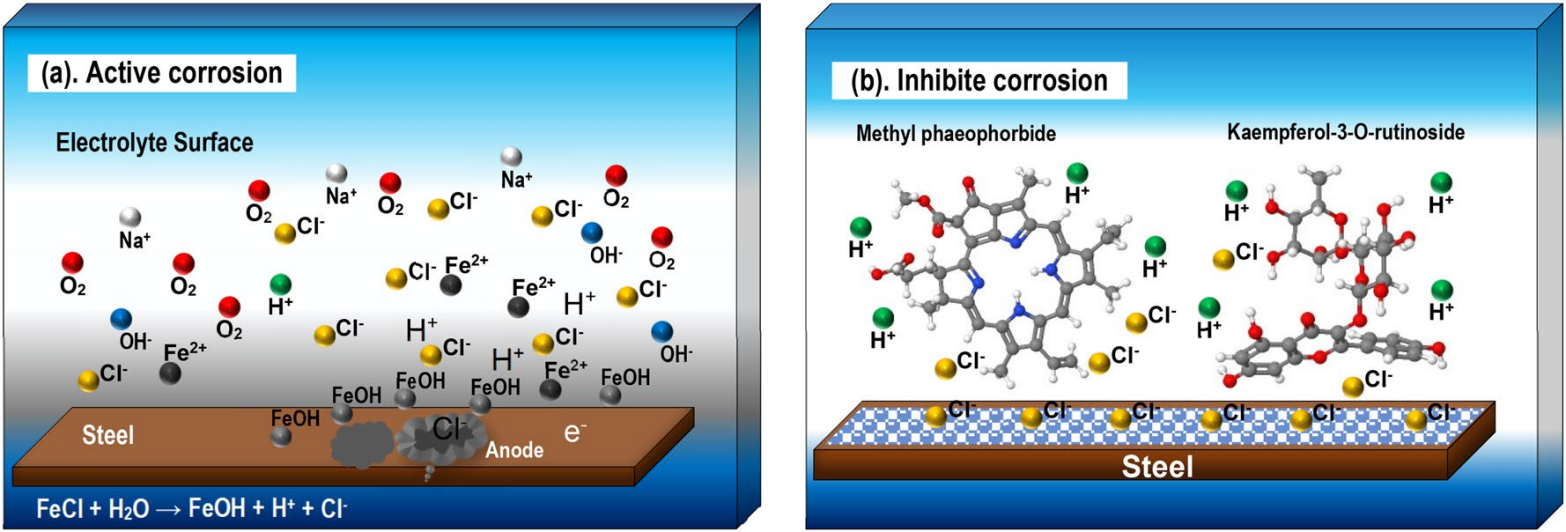
Schematic of (**a**) active corrosion pit and (**b**) corrosion inhibition by active extract compounds on steel in seawater environment.

The corrosion rate data demonstrate that the 300 mg L^−1^ *A. vera* extract formulation achieves 83.75% IE on API 5L steel immersed in seawater. Polarization experiments reveal that the extract components work as mixed inhibitors, with a synergistic relationship between concentration and rate of corrosion inhibition. The AC spectrum impedance also indicates that the protective layer forms on the steel surface. Figure 12 depicts the reaction mechanism for corrosion and its prevention for steel surfaces in a seawater solution, which is explained by the reduction and oxidation reactions listed below.

9 $${\text{Fe }} \to {\text{ Fe}}^{{{2} + }} + {\text{ e }}\left( {\text{anodic process}} \right)$$

10 $${\text{2H}}_{{2}} {\text{O }} + {\text{ O}}_{{2}} + {\text{ 4e }} \to {\text{ 4OH}}^{ - } \left( {\text{cathodic process}} \right)$$

11 $${\text{NaCl }} \to {\text{ Na}}^{ + } + {\text{ Cl}}^{ - }$$

12 $${\text{FeCl }} + {\text{ H}}_{{2}} {\text{O }} \to {\text{ FeOH }} + {\text{ H}}^{ + } + {\text{ Cl}}^{ - } \left( {{\text{hydrolysis}}} \right)$$

Iron complexes formed in the solution when the inhibitor (*A. vera*) was added to a seawater formulation. The formation of complex iron begins with the diffusion of active substances towards the metal surface during exposure to steel in a solution containing the extract. Furthermore, on the steel surface, the active substance reacts with iron to form complex iron. Fe^2+^ ions react with OH^−^ to form Fe(OH)_2_ as a protective layer in the cathodic area according to reaction Eq. (13)^76^:

13 $${\text{Fe}}^{{{2} + }} + {\text{ 2OH}}^{ - } \to {\text{ Fe}}\left( {{\text{OH}}} \right)_{{2}} \downarrow$$

Therefore, complex Fe^2+^ and Fe(OH)_2_ produce the protective layer, which has a synergistic effect. Additionally, organic molecules that have heteroatoms, electronegative functional groups, electron double bonds, and aromatic rings that form bonds with metal atoms are typically the ones that prevent corrosion^17,18,28^. It is known that organic inhibitors work by first substituting inhibitor molecules for water molecules, which starts the metal adsorption process. In different ways, sometimes concurrently, organic corrosion inhibitors can slow down corrosion when in contact with corrosive media. Because experimental conditions can alter the mechanism, it is challenging to identify a mechanism precisely.

Corrosion inhibition through combined physisorption-chemisorption involves the simultaneous action of inhibitors that use both methods to protect metal surfaces from corrosion^57^. Physisorption inhibitors can form a protective layer on metal surfaces through low physical interaction like hydrogen bonds or Van der Waals forces^17^. In contrast, chemisorption inhibitors generate stronger chemical interactions with metal surfaces, resulting in a more stable and long-lasting protective layer^77^. Physisorption inhibitors can be adsorbed on metal surfaces initially by adsorbing chloride ions (Cl^−^) via the electrostatic relationship with the steel surface (Fe^2+^). Positive surface charge facilitates anion adsorption, while negative charge enhances cation adsorption. However, organic compounds in solution are quickly protonated and changed to cations. Protonation occurred in both simulated seawater and *A. vera*. Protonated *A. vera* cannot directly adhere to the positively charged steel surface due to the repulsive interaction between the active substance and the steel surface, but it can adhere to the steel surface via electrostatic interactions between Cl^−^ and protonated *A. vera*. Compounds containing nitrogen frequently exhibit physisorption^78^. Some groups in most extracts, such as ester groups, aromatic groups, and -NH, are rapidly protonated in aggressive media^58^.

Chemisorption occurs when electrostatic interactions protonate the Cl^−^ and *A. vera* ionic interactions. Furthermore, heteroatoms (S, N, and O) and other double bonds in the *A. vera* molecule can have several free electron pairs in the neutral state, which can cause d-orbital vacancies in Fe metal, resulting in coordination bonds like the Fe–N and Fe–S, which considerably improve chemical absorption. The adsorption of aromatic and ester groups at the anodic area on the surface prevents API 5L steel from dissolution. Furthermore, the inhibitor molecules' P-electrons adhere to the steel's anodic surface, reducing corrosion to the iron metal on the anode area. As a result, the electrochemical corrosion process of API 5L steel was reduced by *A. vera* extract^48^. When both types of inhibitors are present, they can work synergistically to provide better corrosion protection^28^.

## Conclusion

Based on the findings of the analysis and evaluation of the performance of *A. vera* extracts as green inhibitors on API 5L steel in seawater environment, the conclusion points obtained can be described as follows: The electrochemical analysis indicates that *A. vera* extract can successfully reduce the corrosion of API 5L steel in seawater environments by adsorbing on metal surfaces. The PDP analysis achieved an optimal corrosion efficiency of around 83.75%, and the EIS analysis achieved an optimum of 88.60% with an inhibitor concentration of 300 mg L^−1^ at 310 K.

- The analysis results reveal that *A. vera* extract has a mixed-type inhibitor and tends to be an exothermic reaction.
- The higher the concentration of *A. vera* extract, the greater the activation energy (E_a_), with the highest activation energy being 48.24 kJ mol^−1^ for the concentration of 300 mg L^−1^.
- Increasing the temperature and exposure time tends to reduce the corrosion inhibition efficiency (IE) values, and the optimum exposure time was obtained at 30 min with 88.34% IE by a concentration of 300 mg L^−1^ at 300 K.
- The surface morphology of the steel treated with *A. vera* extract was smoother, presenting the efficacy of the active components in the *A. vera* extract.
- The dominant components of active substance in *A. vera* extract strongly suspected to be responsible for the inhibition based on LCMS analysis are *methyl phaeophorbide* and *kaempferol-3-O-rutinoside*.
- The corrosion inhibition mechanism by extract was initiated by adsorbing the bioactive component onto the steel surface, forming a protective layer of iron (physisorption-chemisorption process).
- This unveiling investigation discovered that *A. vera* extract has the potential to be a green corrosion inhibitor in seawater environments.

## Data Availability

The corresponding author has all the data connected to this work. All data or inquiries connected to this study can be acquired by contacting the corresponding author.

## References

[1] Xu Y, Huang Y, Cai F, Lu D, Wang X (2022). Study on corrosion behavior and mechanism of AISI 4135 steel in marine environments based on field exposure experiment. Sci. Total Environ..

[2] Refait P, Grolleau AM, Jeannin M, Rémazeilles C, Sabot R (2020). Corrosion of carbon steel in marine environments: Role of the corrosion product layer. Corros. Mater. Degrad..

[3] Gudić S, Vrsalović L, Matošin A, Krolo J, Oguzie EE, Nagode A (2023). Corrosion behavior of stainless steel in seawater in the presence of sulfide. Appl. Sci..

[4] Xu Y, Zhou Q, Liu L, Zhang Q, Song S, Huang Y (2020). Exploring the corrosion performances of carbon steel in flowing natural sea water and synthetic sea waters. Corros. Eng. Sci. Technol..

[5] Xu P, Zhao M, Fu X, Zhao C (2022). Effect of chloride ions on the corrosion behavior of carbon steel in an iron bacteria system. RSC Adv..

[6] Paul S (2010). Estimation of corrosion rate of mild steel in sea water and application of genetic algorithms to find minimum corrosion rate. Can. Metall. Q..

[7] Royani A (2023). Enhancing the corrosion inhibition performance of *Tinospora cordifolia* extract using different fractions of methanol solvent on carbon steel corrosion in a seawater-simulated solution. Appl. Surf. Sci. Adv..

[8] Verma C, Ebenso EE, Bahadur I, Quraishi MA (2018). An overview on plant extracts as environmental sustainable and green corrosion inhibitors for metals and alloys in aggressive corrosive media. J. Mol. Liq..

[9] Udunwa DI, Onukwuli OD, Menkiti MC, Nwanonenyi SC, Ezekannagha CB, Aniagor CO (2024). Experimental, computational, and theoretical studies on the corrosion inhibition potential of green *Gongronema latifolium* extract as corrosion inhibitor for aluminum alloy in HCl solutions. J. Mol. Struct..

[10] Martinez-Gonzalez JJ, Tello-Salgado I, Avilés-Flores M, Landeros-Martínez LL, Flores-De los Ríos JP, Gonzalez-Rodriguez JG (2023). Green corrosion and DFT studies of *Ustilago maydis* extract for carbon steel in sulfuric acid. J. Mol. Struct..

[11] Sowmyashree AS (2023). Potential sustainable electrochemical corrosion inhibition study of *Citrus limetta* on mild steel surface in aggressive acidic media. J. Mater. Res. Technol..

[12] Zhou Z, Min X, Wan S, Liu J, Liao B, Guo X (2023). A novel green corrosion inhibitor extracted from waste feverfew root for carbon steel in H_2_SO_4_ solution. Results Eng..

[13] Dehghani A, Mostafatabar AH, Ramezanzadeh B (2023). Synergistic anticorrosion effect of *Brassica hirta* phytoconstituents and cerium ions on mild steel in saline media: Surface and electrochemical evaluations. Colloids Surf. A Physicochem. Eng. Asp..

[14] Royani A, Hanafi M, Manaf A (2022). Prospect of plant extracts as eco-friendly biocides for microbiologically influenced corrosion: A review. Int. J. Corros. Scale Inhib..

[15] Ali IH, Idris AM, Suliman MHA (2019). Evaluation of leaf and bark extracts of *Acacia tortilis* as corrosion inhibitors for mild steel in seawater: Experimental and studies. Int. J. Electrochem. Sci..

[16] Haddadi SA, Alibakhshi E, Bahlakeh G, Ramezanzadeh B, Mahdavian M (2019). A detailed atomic level computational and electrochemical exploration of the *Juglans regia* green fruit shell extract as a sustainable and highly efficient green corrosion inhibitor for mild steel in 3.5 wt% NaCl solution. J. Mol. Liq..

[17] Miralrio A, Vázquez AE (2020). Plant extracts as green corrosion inhibitors for different metal surfaces and corrosive media: A review. Processes.

[18] Ben Harb M, Abubshait S, Etteyeb N, Kamoun M, Dhouib A (2020). Olive leaf extract as a green corrosion inhibitor of reinforced concrete contaminated with seawater. Arab. J. Chem..

[19] Barbouchi M, Benzidia B, Aouidate A, Ghaleb A, El Idrissi M, Choukrad M (2020). Theoretical modeling and experimental studies of Terebinth extracts as green corrosion inhibitor for iron in 3% NaCl medium. J. King Saud Univ. Sci..

[20] Bahlakeh G, Dehghani A, Ramezanzadeh B, Ramezanzadeh M (2019). Highly effective mild steel corrosion inhibition in 1 M HCl solution by novel green aqueous mustard seed extract: Experimental, electronic-scale DFT and atomic-scale MC/MD explorations. J. Mol. Liq..

[21] Medupin RO, Ukoba KO, Yoro KO, Jen TC (2023). Sustainable approach for corrosion control in mild steel using plant-based inhibitors: A review. Mater. Today Sustain..

[22] Ikhmal WMKWM, Maria MFM, Rafizah WAW, Norsani WNWM, Sabri MGM (2019). Corrosion inhibition of mild steel in seawater through green approach using *Leucaena leucocephala* leaves extract. Int. J. Corros. Scale Inhib..

[23] Zakeri A, Bahmani E, Aghdam ASR (2022). Plant extracts as sustainable and green corrosion inhibitors for protection of ferrous metals in corrosive media: A mini review. Corros. Commun..

[24] Vorobyova V, Skiba M, Andrey K (2022). Tomato pomace extract as a novel corrosion inhibitor for the steel in industrial media: The role of chemical transformation of the extract and proinhibition effect. J. Mol. Struct..

[25] Haddadi SA, Keramatinia M, Ramezanzadeh M, Ramezanzadeh B (2022). Detailed experimental investigation of the highly active corrosion inhibitive green molecules based on zinc cations/*Nepeta pogonosperma* extract and toward the corrosion mitigation of mild steel in the saline solution. Colloids Surf. A Physicochem. Eng. Asp..

[26] Hossein Jafari Mofidabadi A, Dehghani A, Ramezanzadeh B (2022). Investigating the effectiveness of watermelon extract-zinc ions for steel alloy corrosion mitigation in sodium chloride solution. J. Mol. Liq..

[27] Vorobyova V, Skiba M, Gnatko E (2023). Agri-food wastes extract as sustainable-green inhibitors corrosion of steel in sodium chloride solution: A close look at the mechanism of inhibiting action. S. Afr. J. Chem. Eng..

[28] Luo X (2023). Synthesis of natural glucomannan derivative as a highly-efficient green inhibitor for mild steel in the simulated seawater. J. Ind. Eng. Chem..

[29] Liu Y (2023). Corrosion behaviour of hot-rolled 316L stainless steel-A6 carbon steel composite steel plate for marine environment. J. Mater. Res. Technol..

[30] Lv M, Du M, Li Z (2022). Investigation of mixed species biofilm on corrosion of X65 steel in seawater environment. Bioelectrochemistry.

[31] Taştan Z, Alp Avci G, Uysal Kilic T, Avci E (2024). In vitro evaluation of the biological activity potential of *Aloe vera* gel: Antioxidant activity and cytotoxic effects in Hepg2 and L929. J. Microbiol. Biotechnol. Food Sci..

[32] Kumar S (2017). Evaluating antimicrobial activity of *Aloe vera* plant extract in human life. Biomed. J. Sci. Tech. Res..

[33] Hossen MM, Hossain ML, Mitra K, Hossain B, Bithi UH, Uddin MN (2022). Phytochemicals and in-vitro antioxidant activity analysis of *Aloe vera* by-products (skin) in different solvent extract. J. Agric. Food Res..

[34] López Z (2017). Antioxidant and cytotoxicological effects of *Aloe vera* food supplements. J. Food Qual..

[35] Khiya Z (2019). Valorization of the *Salvia officinalis* L. of the Morocco bioactive extracts: Phytochemistry, antioxidant activity and corrosion inhibition. J. King Saud Univ. Sci..

[36] Benzidia B, Barbouchi M, Rehioui M, Hammouch H, Erramli H, Hajjaji N (2023). *Aloe vera* mucilage as an eco-friendly corrosion inhibitor for bronze in chloride media: Combining experimental and theoretical researches. J. King Saud Univ. Sci..

[37] Mashooque S, Kumar M, Unar IN (2022). Effect of *Aloe vera* extract asgreen corrosion inhibitor on medium carbon steel in sulphuric acid environment. Pak. J. Anal. Environ. Chem..

[38] Sobhy MA, Mahross MH, Abbas MA, El Zomrawy A (2021). Evaluation of *Aloe vera* gel extract as eco-friendly corrosion inhibitor for carbon steel in 1.0 M HCl. Egypt. J. Chem..

[39] Šćepanović J, Herenda S, Radonjić D, Vuksanović D (2019). Investigation of inhibitory effect of the *Aloe vera* extract on corrosion of aluminium alloys. Bull. Chem. Technol. Bosnia Herzegovina.

[40] Ndibe M, Menkiti M, Ijomah M, Onukwuli D, Ejikeme P (2018). Acid extract of aloe vera as inhibitor for the corrosion of mild steel in acidic media. Environ. Eng. Manag. J..

[41] Kumar H, Yadav V (2018). *Aloe vera* L. as green corrosion inhibitor for mild steel in 5.0 M hydrochloric acid solution. Asian J. Chem..

[42] Royani A (2023). Isolation and identification of bioactive compounds from *Tinospora cordifolia* stem extracts as antibacterial materials in seawater environments. Arab. J. Chem..

[43] Ferkous H (2024). A comparative study of novel synthesized sulfamide compounds: Electrochemical, morphological, XPS, and theoretical investigations on copper corrosion inhibition in 1.0 M HCl. J. Mol. Liq..

[44] Minagalavar RL, Rajappa SK, Rathod MR, Sajjan AM (2024). Ferrous metal corrosion studies in presence of eco-friendly *Acacia melanoxylon* leaves extract in 1M HCl condition. Inorg. Chem. Commun..

[45] Vashi RT, Chaudhari HG (2017). The study of *Aloe-vera* gel extract as green corrosion inhibitor for mild steel in acetic acid. Int. J. Innov. Res. Sci..

[46] Cherrad S (2022). Cupressus arizonica fruit essential oil: A novel green inhibitor for acid corrosion of carbon steel: *Cupressus arizonica* fruit essential oil. Arab. J. Chem..

[47] Fernandes CM (2019). Ircinia strobilina crude extract as corrosion inhibitor for mild steel in acid medium. Electrochim. Acta.

[48] Ayoola AA (2020). Inhibitive corrosion performance of the eco-friendly *Aloe vera* in acidic media of mild and stainless steels. J. Bio- Tribo-Corros..

[49] Rehioui M (2024). 1,2,4-triazole-5-thione derivative for inhibiting carbon steel corrosion in 1M HCl: Synthesis, electrochemical, SEM/EDX, DFT, and MD investigations. J. Mol. Struct..

[50] Rabizadeh T, Asl SK (2019). Casein as a natural protein to inhibit the corrosion of mild steel in HCl solution. J. Mol. Liq..

[51] Benzidia B, Barbouchi M, Hsissou R, Zouarhi M, Erramli H, Hajjaji N (2022). A combined experimental and theoretical study of green corrosion inhibition of bronze B66 in 3% NaCl solution by *Aloe saponaria* (syn. *Aloe maculata*) tannin extract. Curr. Res. Green Sustain. Chem..

[52] Rehioui M (2024). 1,2,4-triazole-5-thione derivative for inhibiting carbon steel corrosion in 1M HCl: Synthesis, electrochemical, SEM/EDX, DFT, and MD investigations. J. Mol. Struct..

[53] Muthukrishnan P, Jeyaprabha B, Prakash P (2017). Adsorption and corrosion inhibiting behavior of *Lannea coromandelica* leaf extract on mild steel corrosion. Arab. J. Chem..

[54] Wang Q (2023). Experimental and theoretical insights into *Oxalis corniculata* L. extract as a sustainable and eco–friendly corrosion inhibitor for carbon steel in acidic environments. Mater. Chem. Phys..

[55] Maizia R (2023). Experimental assessment and molecular-level exploration of the mechanism of action of Nettle (*Urtica dioica* L.) plant extract as an eco-friendly corrosion inhibitor for X38 mild steel in sulfuric acidic medium. Arab. J. Chem..

[56] Zhu L (2021). Inhibitive effect of different solvent fractions of bamboo shoots extract on the corrosion of mild steel in 0.5 mol/L H_2_SO_4_ solution. J. Mol. Struct..

[57] Singh AK, Mohapatra S, Pani B (2016). Corrosion inhibition effect of *Aloe vera* gel: Gravimetric and electrochemical study. J. Ind. Eng. Chem..

[58] Haque J, Srivastava V, Chauhan DS, Quraishi MA, Madhan Kumar A, Lgaz H (2020). Electrochemical and surface studies on chemically modified glucose derivatives as environmentally benign corrosion inhibitors. Sustain. Chem. Pharm..

[59] Zhang W, Ma Y, Chen L, Wang LJ, Wu YC, Li HJ (2020). Aloe polysaccharide as an eco-friendly corrosion inhibitor for mild steel in simulated acidic oilfield water: Experimental and theoretical approaches. J. Mol. Liq..

[60] Abdullah HA, Anaee RA, Khadom AA, Abd AT, Malik AH, Kadhim MM (2023). Experimental and theoretical assessments of the chamomile flower extract as a green corrosion inhibitor for aluminum in artificial seawater. Results Chem..

[61] Royani A, Hanafi M, Haldhar R, Manaf A (2024). Evaluation of *Morinda citrifolia* extract as sustainable inhibitor for mild steel in saline environment. J. Eng. Res..

[62] Alamry KA, Khan A, Aslam J, Hussein MA, Aslam R (2023). Corrosion inhibition of mild steel in hydrochloric acid solution by the expired Ampicillin drug. Sci. Rep..

[63] Alaneme KK, Olusegun SJ, Adelowo OT (2016). Corrosion inhibition and adsorption mechanism studies of *Hunteria umbellata* seed husk extracts on mild steel immersed in acidic solutions. Alex. Eng. J..

[64] Moustafa AHE, Abdel-Rahman HH, Awad MK, Naby AANA, Seleim SM (2022). Molecular dynamic simulation studies and surface characterization of carbon steel corrosion with changing green inhibitors concentrations and temperatures. Alex. Eng. J..

[65] Ajayi, O., Everitt, N. M. & Voisey, K. T. Evaluation of inulin and aloe vera as green corrosion inhibitors for mild steel in 15 % HCl. In *European Federation of Corrosion Congress*, 1–15 (2016) https://nottingham-repository.worktribe.com/output/802616.

[66] Pankaj G, Gargi J (2014). Corrosion inhibition by *Aloe barbadensis* (*Aloe vera*) extract as green inhibitor for mild steel in HNO_3_. Int. J. Sci. Res. Rev..

[67] Gadow HS, Motawea MM (2017). Investigation of the corrosion inhibition of carbon steel in hydrochloric acid solution by using ginger roots extract. RSC Adv..

[68] Xiong L, Wang P, He Z, Chen Q, Pu J, Zhang R (2021). Corrosion behaviors of Q235 carbon steel under imidazoline derivatives as corrosion inhibitors: Experimental and computational investigations. Arab. J. Chem..

[69] Derbe T, Yilma B (2015). Investigation of the anti-corrosion activities of *Aloe vera* extract on iron metal sheets. J. Nat. Sci. Res..

[70] Unueroh U, Ebebeinwe AO (2022). Investigating the effect of *Aloe-vera* extract on the corrosion inhibition of medium carbon steel in seawater. Int. J. Mater. Eng. Technol..

[71] Benzidia B, Barbouchi M, Hammouch H, Erramli H, Hajjaji N (2023). The exploitation of *Aloe saponaria* (syn. *Aloe maculata*) as a potential green corrosion inhibitor for bronze in a neutral chloride environment. Biointerface Res. Appl. Chem..

[72] Bodian M, Keinde D, Hannawi K, Fall M, Darquennes A (2024). Evaluation of the inhibitory gel *Aloe vera* against corrosion of reinforcement concrete in NaCl medium. Mater. Sci. Appl..

[73] Alanazi KD (2024). Thermodynamic, chemical, and electrochemical studies of *Aloe ferox* Mill extract as a naturally developing copper corrosion inhibitor in HCl solution. Sci. Rep..

[74] Lawal A, Musa GK, Mashi AL (2024). Exploring nymphaeaceae and *Aloe barbadenis* extracts as corrosion inhibitors for mild steel: Electrochemical assessment in acidic medium (H_2_SO_4_). Jabirian J. Biointerface Res. Pharm. Appl. Chem..

[75] Othman NK, Yahya S, Ismail MC (2019). Corrosion inhibition of steel in 3.5% NaCl by rice straw extract. J. Ind. Eng. Chem..

[76] Veedu KK, Mohan S, Somappa SB, Gopalan NK (2022). Eco-friendly anticorrosive epoxy coating from Ixora leaf extract: A promising solution for steel protection in marine environment. J. Clean. Prod..

[77] Noor EA, Al-moubaraki AH (2008). Thermodynamic study of metal corrosion and inhibitor adsorption processes in mild steel/1-methyl-4 [4ʹ(-X)-styryl pyridinium iodides/hydrochloric acid systems. Mater. Chem. Phys..

[78] Faiz M, Zahari A, Awang K, Hussin H (2020). Corrosion inhibition on mild steel in 1 M HCl solution by: *Cryptocarya nigra* extracts and three of its constituents (alkaloids). RSC Adv..

